# Supplementary material for: Massively parallel fabrication of crack-defined gold break junctions featuring sub-3 nm gaps for molecular devices
Source: Nat Commun. 2018 Aug 24;9:3433. doi: 10.1038/s41467-018-05785-2 (PMC6109151; doi:10.1038/s41467-018-05785-2)
Supplement: Supplementary file 1 — Supplementary Information [file 41467_2018_5785_MOESM1_ESM.pdf]

Supplementary information

Massively parallel fabrication of crack-defined gold break junctions featuring sub-3 nm gaps for molecular devices

Valentin Dubois[1], Shyamprasad N. Raja[1,+], Pascal Gehring[2, +], Sabina Caneva[2], Herre S. J. van de Zant[2], Frank Niklaus[1,] and Göran Stemme[1,]**

[1]Department of Micro and Nanosystems, School of Electrical Engineering, KTH Royal Institute of Technology, SE-10044 Stockholm, Sweden

[2]Kavli Institute of Nanoscience, Delft University of Technology, Lorentzweg 1, 2628 CJ Delft, The Netherlands

[+] These authors contributed equally to this work.

*E-mail: goran.stemme@eecs.kth.se

*E-mail: frank.niklaus@eecs.kth.se

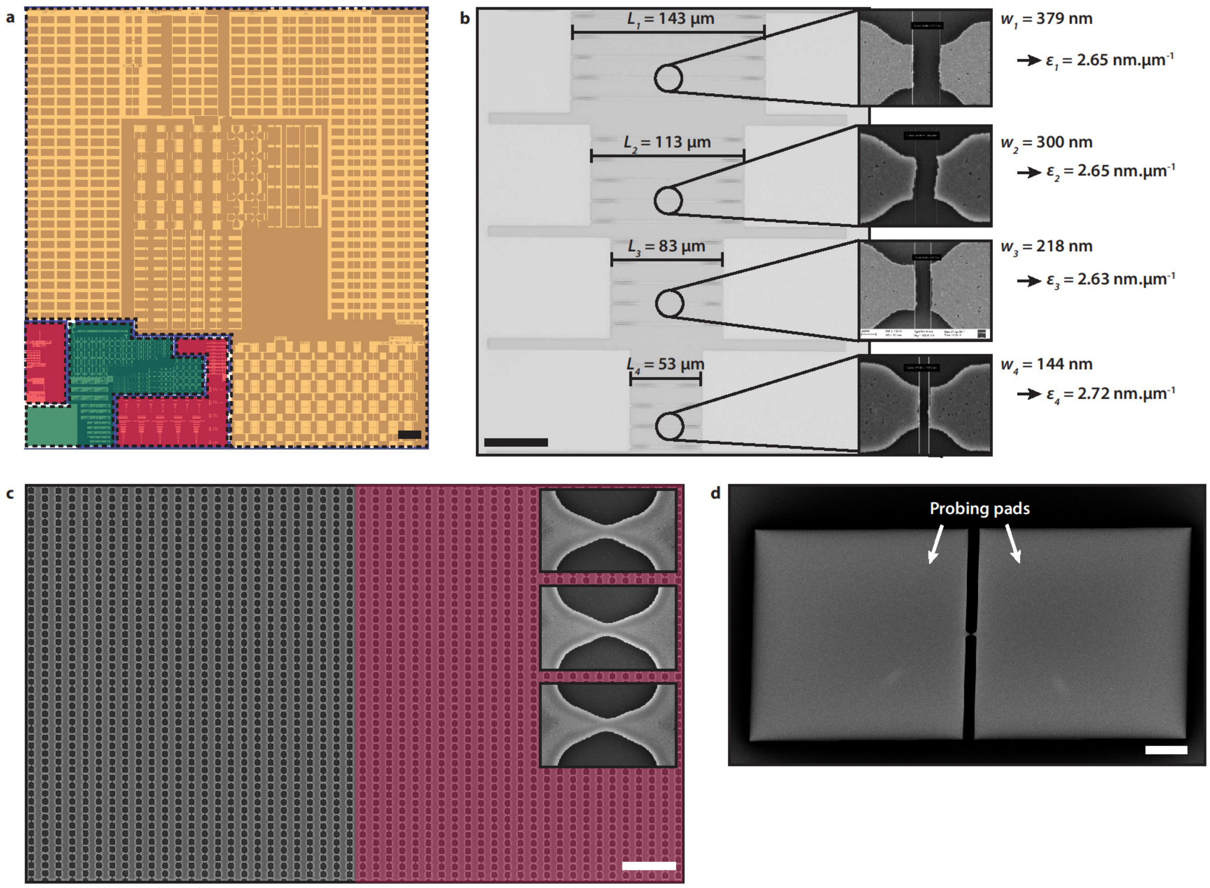

Supplementary Figure 1. Details of the design of each of the 100 unit cells fabricated on the wafer, including optical microscope and SEM images of selected structures. (**a**) Schematic top view drawing of a cell showing the four main areas. Most of the area of each cell is occupied by electrically probable single break junctions (orange overlay, see **d** for an example of a probable junction). Other areas contain process test structures (green overlay) and high-density arrays of break junctions (red overlay) for e.g. measuring the elastic strain (see **b**) or estimating the yield of fracture of some selected designs (see **c**). (**b**) Optical microscope image of an array of bridges with variations in *L* used to determine the elastic strain *ε* of the TiN layer. The bridges have lengths spanning 90 µm from 53 µm to 143 µm with steps of 30 µm. Five repetitions of each bridge design are included. An average elastic strain of 2.66 nm.µm^{-1} (rounded up to 2.7 in the main text) is found for the TiN deposited on this wafer. (**c**) SEM image of an array of 2500 junctions with a false color overlay highlighting the 1250 junctions inspected using SEM imaging. Of the 1250 examined junctions, only 3 bridges were found uncracked, thus indicating a yield of fracture exceeding 99.7%. The SEM images of the 3 uncracked bridges are shown on the top right corner. For the bridge design in this array, a single junction fits in an area of 4.8 µm × 3 µm = 14.4 µm^2. Thus, the calculated density is: $(14.4\cdot10^{-8})^{-1}$ ≈ 6,944,440 junctions per cm^2, allowing for $\pi \times 5^2 \times (14.4 \times 10^{-8})^{-1}$ ≈ 545,415,390 junctions per 100 mm diameter wafer. (**d**) SEM image of an electrically probable junction with 100 µm × 100 µm sized probing pads connected to each electrode. We chose this area-consuming approach with two dedicated probing pads per junction to minimize the risk for accidental electrical damage of junctions. Scale bar is 500 µm in **a**, 50 µm in **b**, and 20 µm in **c** and **d**.

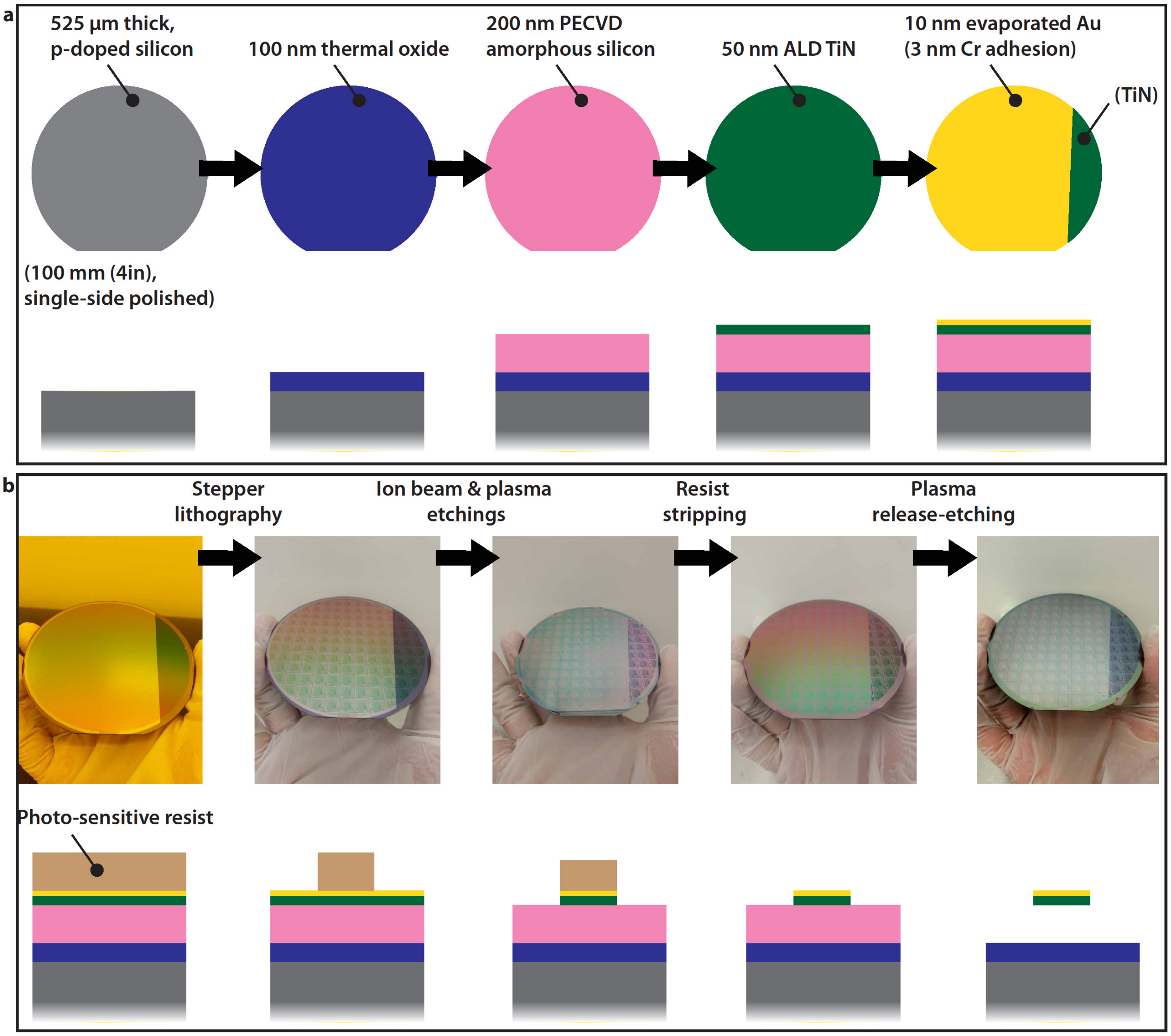

Supplementary Figure 2. Description of the wafer preparation and fabrication of crack-defined break junctions. (**a**) Schematics of the substrate preparation in top and cross-sectional views. The part of the wafer that was not coated with gold on the right side was used for process control and monitoring. (**b**) Photographs of the fabricated wafer in side view and schematics in cross-sectional view for each of the processing steps.

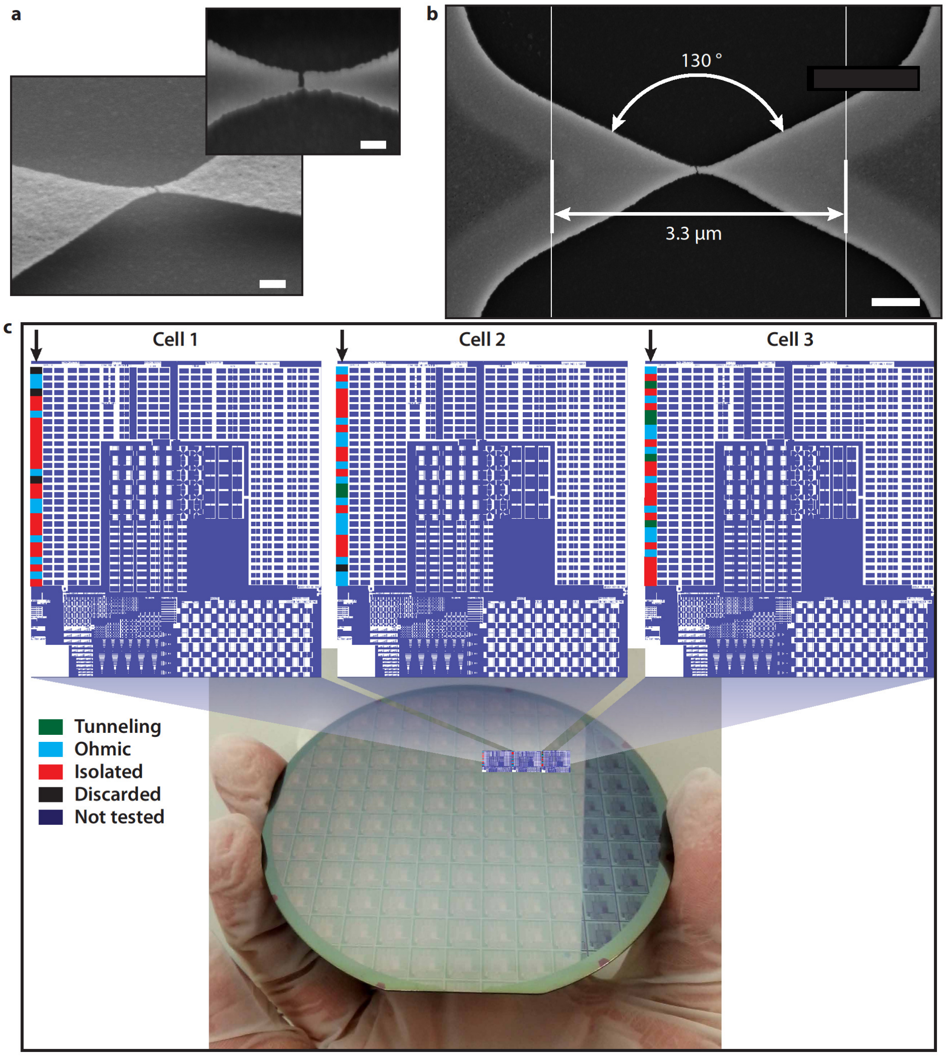

Supplementary Figure 3. Details of the first set of electrically probed break junctions. (**a**) SEM images of a junction in perspective view and (**b**) top view (and inset of **a**). (**c**) Details of the location of 90 probed junctions on the wafer and within each of the three 7 mm × 7 mm sized cells. In this set of experiments, each of the 90 junctions (30 in each cell in the columns indicated by arrows) was first electrically probed to identify the conduction mechanism without prior visual inspection of the junctions. Thereafter, a SEM image of each junction was taken to compare the morphology of the junction with the electrical conduction mechanism of the junction. The *I-V* plots and SEM images of all 30 junctions of Cell 3 are presented in Supplementary Note 1. Scale bar is 200 nm in **a**, 100 nm in the inset of **a**, and 1 µm in **b**.

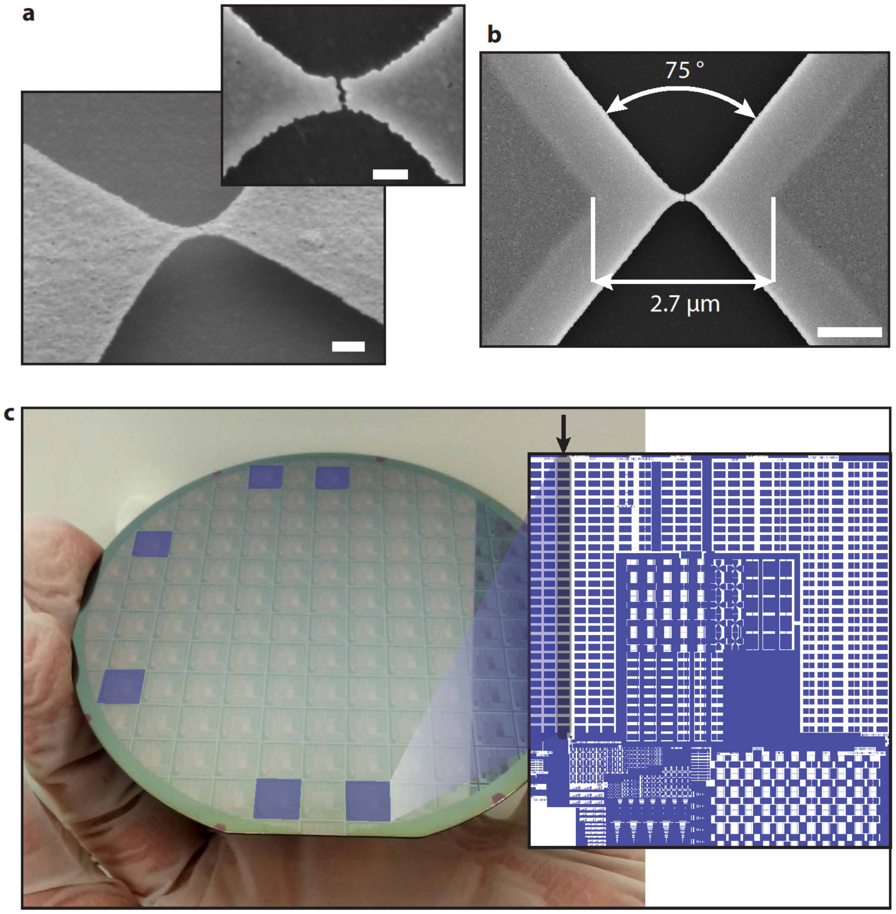

Supplementary Figure 4. Details of the second set of electrically probed break junctions placed over the entire 100 mm diameter wafer. (**a**) SEM images of a junction in perspective view and (**b**) top view (and inset of **a**). (**c**) Details of the location of probed junctions on the wafer and within each cell. In this experiment, the 180 junctions in the six cells highlighted in blue on the wafer were first examined using SEM imaging, and 31 junctions were identified as containing locally pinched ligaments, which is typical for tunneling junctions formed by this method. Then, each of the 31 junctions was electrically probed to discriminate between tunneling junctions and junctions that are electrically connected through the ligaments. It was found that 15 out of the 31 selected junctions (~50%) exhibited tunneling behavior. Scale bar is 200 nm in **a**, 100 nm in the inset of **a**, and 500 nm in **b**.

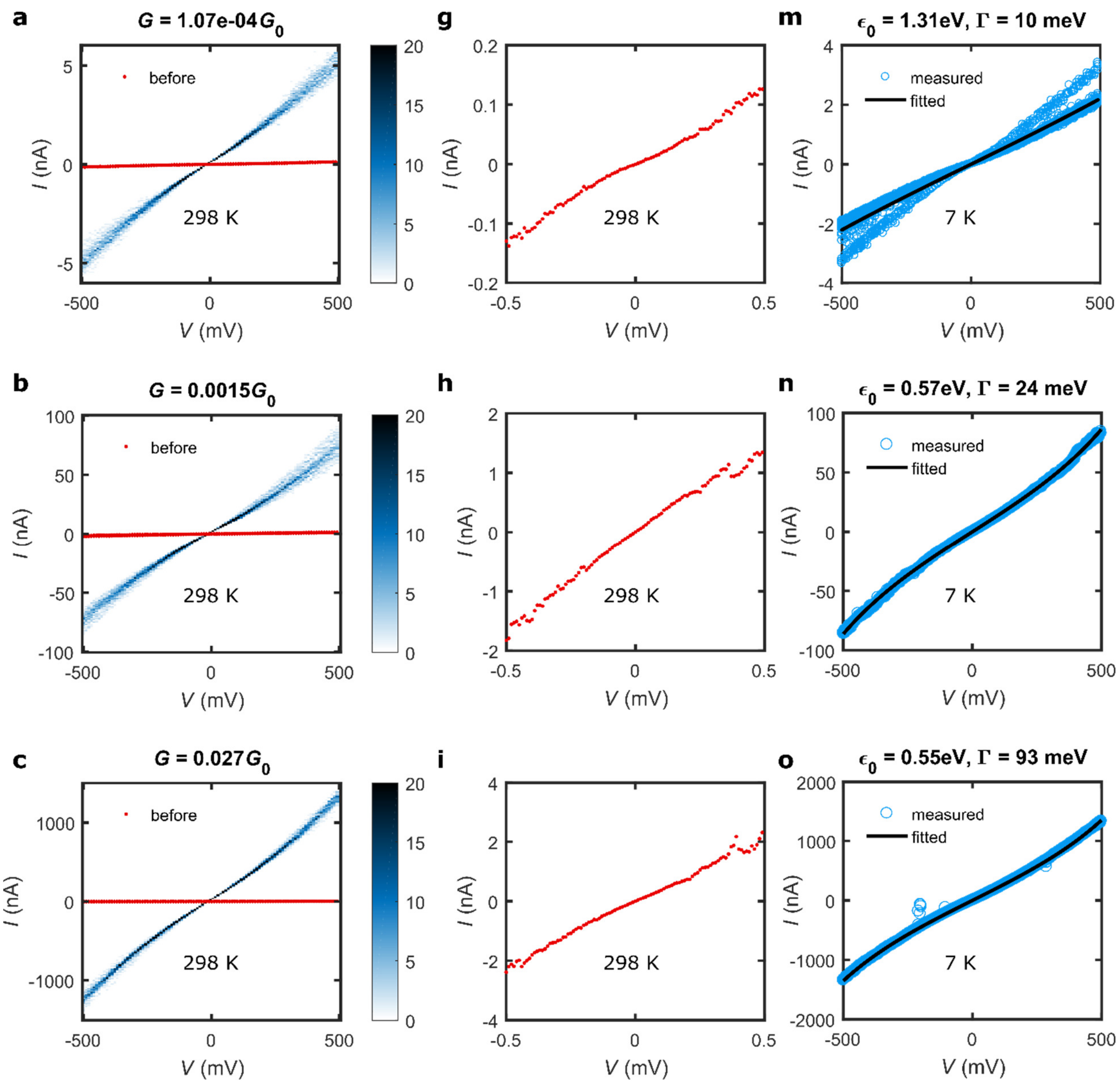

Supplementary Figure 5. (continued in next page)

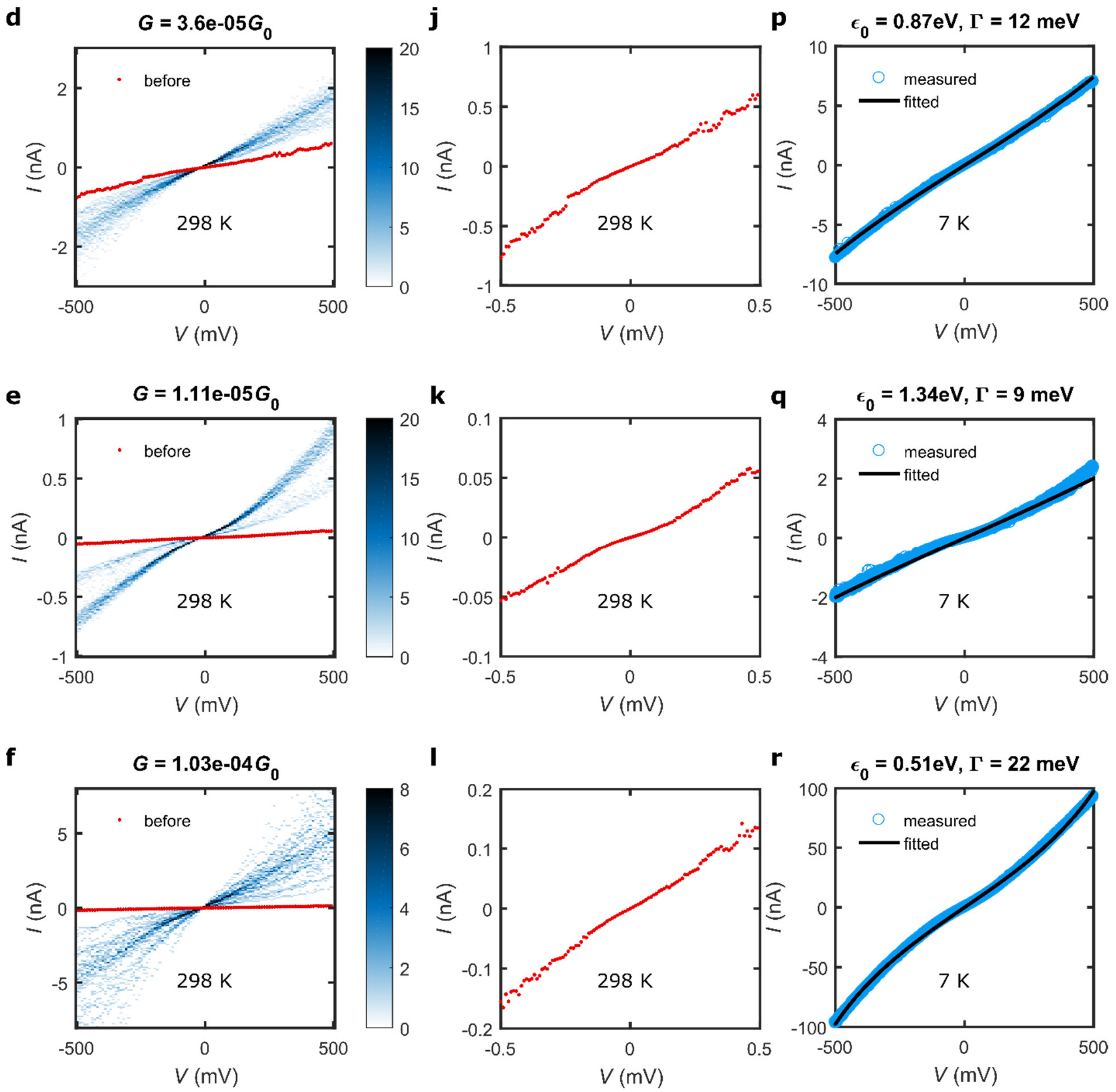

Supplementary Figure 5. The I-V characteristics of six junctions before and after molecule deposition. One device is plotted in each row and three panels are shown for each device: (**a**-**f**) *I*-*V* histogram of an OPE3 junction at 298 K including the *I*-*V* trace before OPE3 deposition in red, and the low bias conductance (*G*) of the OPE3 junction; (**g**-**l**) the same *I*-*V* trace before OPE3 deposition shown separately; (**m**-**r**) *I*-*V* trace of the OPE3 junction at a temperature of 7 K in vacuum, including the fitted curve and parameters for the single-level model.

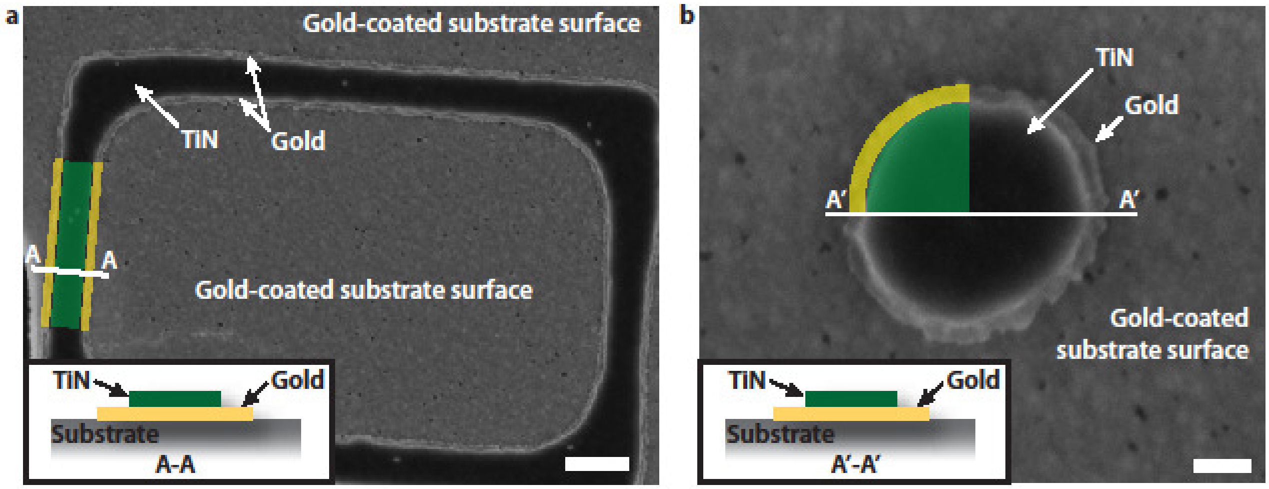

Supplementary Figure 6. Demonstration of the selective wet etching of titanium nitride. (**a**,**b**) Top view SEM images of two fully-released and overturned gold-coated TiN structures with false color overlays to highlight the different materials (green: TiN; yellow: gold) and a schematic cross-sectional view. These images reveal the undercut profile in TiN at the edge of any TiN/gold pattern. The undercut was produced by selective etch of TiN in a solution of SC-1. The presence of a TiN undercut ensures that only the top gold overhangs contribute to the electrical conduction of CDBJs. Nanoscale details of the 20 nm TiN undercut can be seen in **b** of a ~ 300 nm diameter disk. Scale bar is 400 nm in **a**, and 100 nm in **b**.

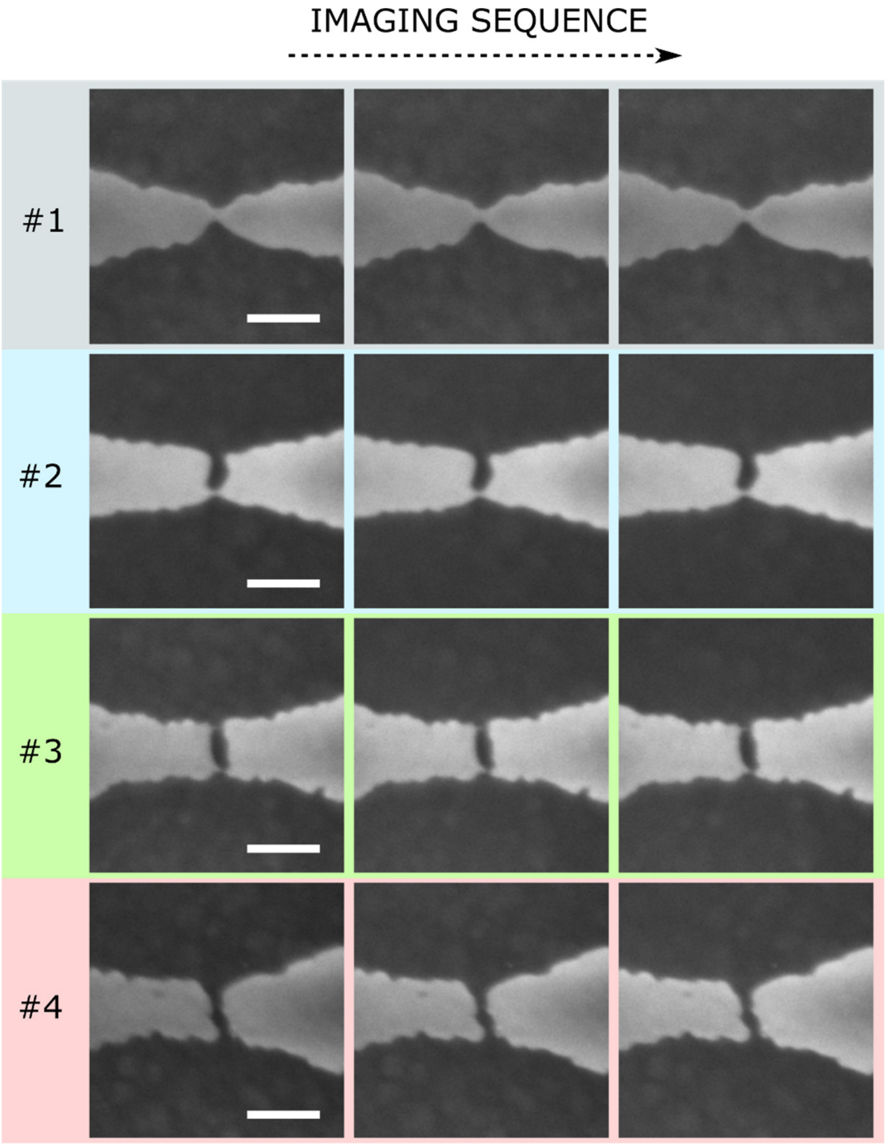

Supplementary Figure 7. Assessing the effect of SEM imaging on junction morphology. The non-invasiveness of SEM imaging can be inferred from the stability of junctions as a series of SEM images of a junction are acquired sequentially. This set of three images each of four junctions was acquired using settings identical to those we have used to correlate the morphology of the junctions to electrical measurements (described in the method section on "Morphological characterization" in the manuscript). Scale bar for all images is 100 nm.

Supplementary Table 1. Fit parameters to the 1-D transmission model for bare tunneling junctions.

#	Sample Type	d [nm]	φ_0 [eV]	R^2
1	From wafer center.<br>*I-V* characterization followed by SEM.<br>$d_\mu = 1.1, d_\sigma = 0.3$<br>$\varphi_{0,\mu} = 2.9, \varphi_{0,\sigma} = 0.6$	1.1	3.5	0.99
2		1.2	2.0	0.97
3		0.8	3.0	0.89
4		1.3	3.0	0.98
5		1.5	2.4	0.95
6		1.0	3.4	0.96
7		0.8	3.2	0.95
8	From wafer periphery.<br>SEM followed by *I-V* characterization<br>$d_\mu = 1.5, d_\sigma = 0.5$<br>$\varphi_{0,\mu} = 2.3, \varphi_{0,\sigma} = 0.5$	1.6	2.5	0.97
9		1.2	2.5	0.96
10		1.0	3.1	0.99
11		1.2	2.6	0.97
12		2.9	1.2	0.94
13		1.3	2.2	0.97
14		1.4	2.7	0.98
15		1.1	2.6	0.99
16		1.6	1.8	0.99
17		1.6	2.1	0.92

Gap width (d) and symmetric potential barrier height ($\varphi_0 = \varphi_L = \varphi_R$), and the coefficient of determination (R^2), obtained from the fits of tunneling *I-V* characteristics of CDBJ showing tunneling *I-V* characteristics to the 1-D transport model described in Methods. The table is divided into two sections: the first 7 devices are from dies at the wafer center, and the remaining 10 devices are from dies at the wafer edge. The mean (subscript μ: d_μ , $\varphi_{0,\mu}$) and standard deviation (subscript σ: d_σ , $\varphi_{0,\sigma}$) of both parameters for each of the two populations are also shown in the table and are very similar. From all these devices we observe that barring one outlier (2.9 nm), the gap width ranges from 0.8 to 1.6 nm, with an average value of 1.3 nm. The average work function is 2.6 eV, which is consistent with the lowering of the work function expected for Au junctions with adsorbed species at the electrode tips. The plots of the *I-V* characteristics including the model fits for the data in rows 1 to 5 of this table correspond to serial numbers 3, 7, 8, 13 and 22 of the preceding series of plots.

Supplementary Table 2. Single-level model parameters for OPE3 molecular junctions extracted from experimental data at 7K.

**Junction**	ε_0 **[eV]**	Γ **[meV]**
a	1.31	10
b	0.57	24
c	0.55	93
d	0.87	12
e	1.34	9
f	0.51	22

The *I*-*V* characteristics at 7K and the single-model fits for these junctions are plotted in the correspondingly named panels **a**–**f** of Supplementary Figure 5.

Supplementary Note 1: I-V characteristics and SEM images of all 30 probed junctions from a cell

A collection of *I*-*V* plots and corresponding SEM micrographs of 30 junctions that are part of Cell 3 (see Supplementary Fig. 3) is included below. To eliminate possible damage and contamination induced by electron beam irradiation, the SEM images were taken after the electrical characterization was completed on all 86 devices.

For the 5 junctions out of 30 which were found to be tunneling, we show the curves fit to asymmetric and symmetric potential barrier 1-D transport models (as described in Supplementary Note 1) and the Fowler-Nordheim (F-N) representation of the *I*-*V* characteristics. In the F-N representation, data points plotted as red crosses fall below the noise threshold of 50 fA for these measurements. In the *I*-*V* plots, we do not show current values below the noise threshold which had negative values of current (only positive bias was applied in these measurements), because of the logarithmic scale used for the *I*-axis in these plots.

<u>Note:</u> because the probed junctions are electrically isolated from the rest of the wafer, charging of the junctions occurs as the electron beam of the SEM scans the area of the junctions. Due to charging, some constrictions may therefore appear wider or narrower, and gaps may appear more oblique than they are in reality.

Scale bar in close-up SEM images: 100 nm

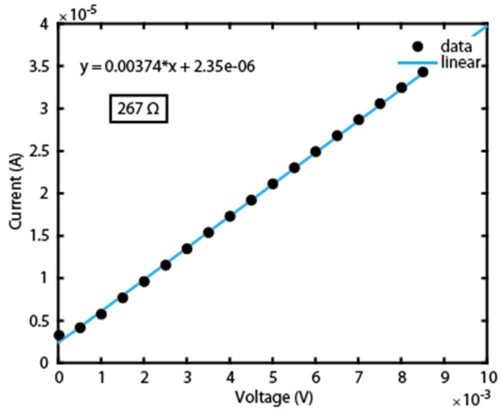

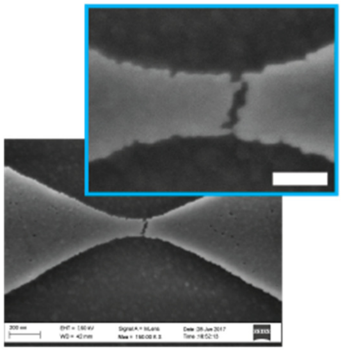

1.
Results from I-V: ohmic

Results from SEM: 2 connected ligaments

Correlation between I-V and SEM: Good

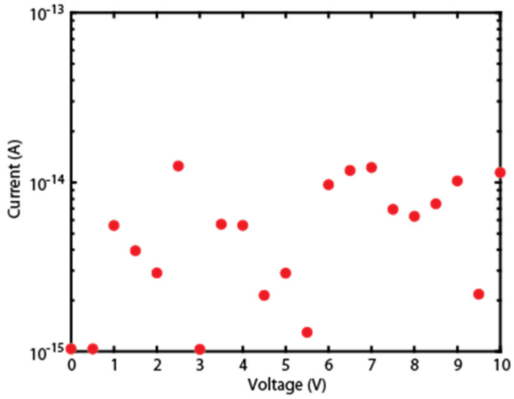

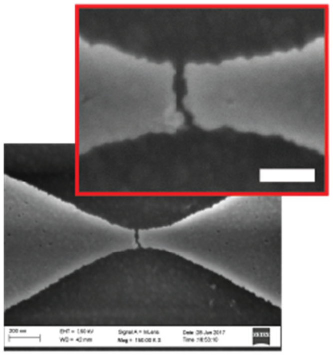

2.
Results from I-V: gap wider than 3 nm

Results from SEM: sub-10 nm gap; traces of contamination

Correlation between I-V and SEM: Good

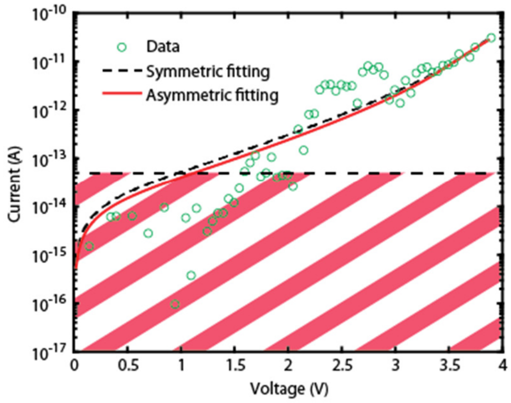

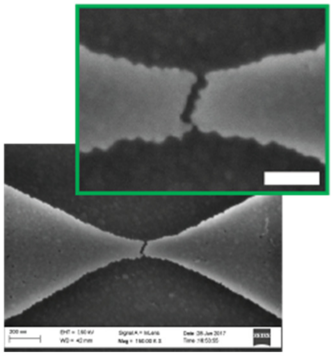

3.
Results from I-V: F-N tunneling

Results from SEM: 1 pinched ligament

Correlation between I-V and SEM: Good

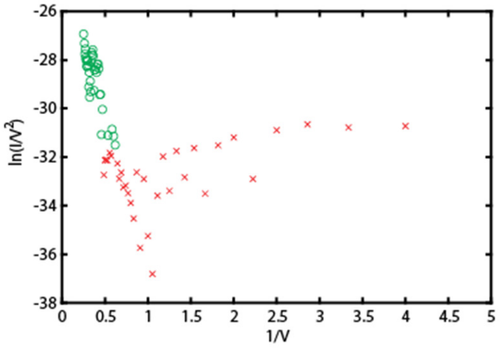

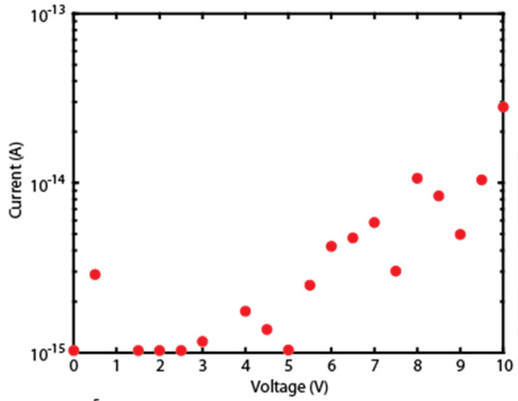

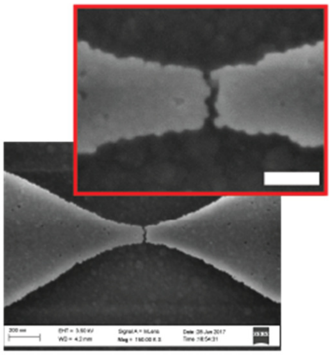

4.
<u>Results from I-V:</u> gap wider than 3 nm

<u>Results from SEM:</u> ~15 nm gap

<u>Correlation between I-V and SEM:</u> Good

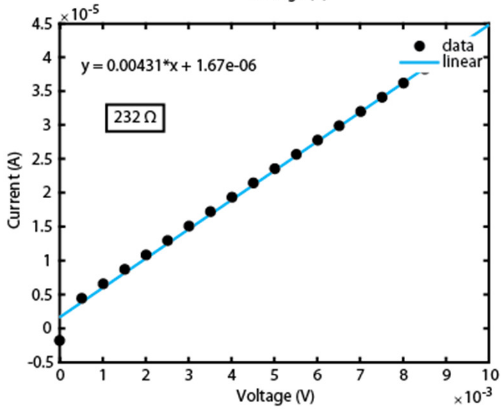

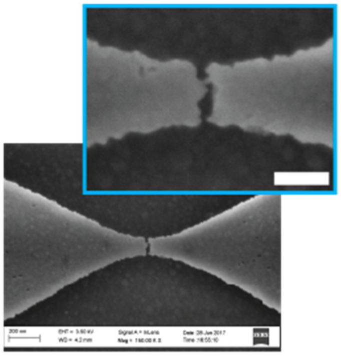

5.
<u>Results from I-V:</u> ohmic

<u>Results from SEM:</u> 1 connected ligament

<u>Correlation between I-V and SEM:</u> Good

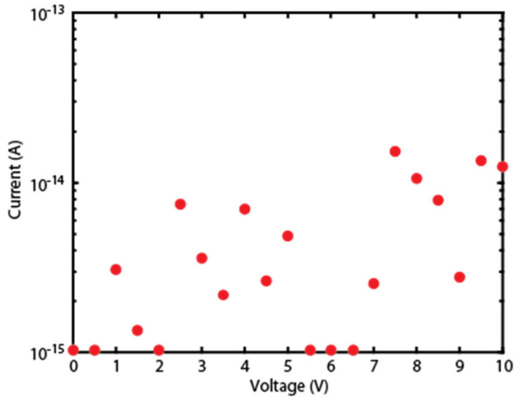

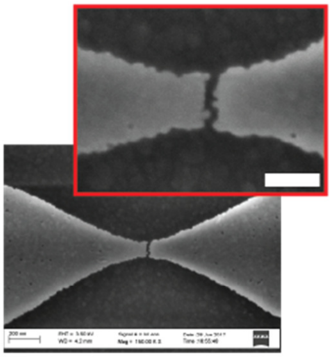

6.
<u>Results from I-V:</u> gap wider than 3 nm

<u>Results from SEM:</u> ~10 nm gap

<u>Correlation between I-V and SEM:</u> Good

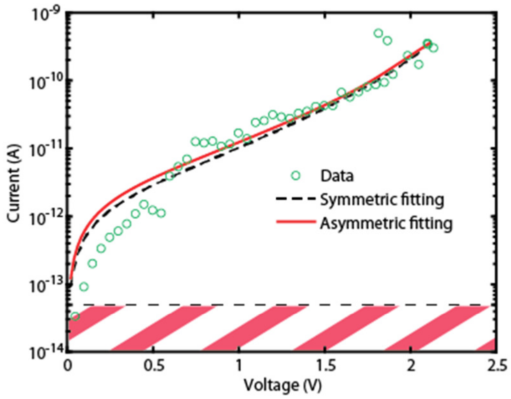

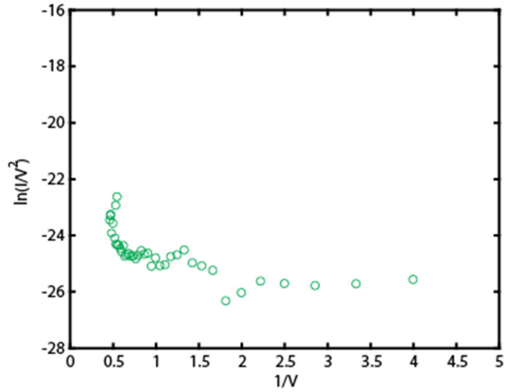

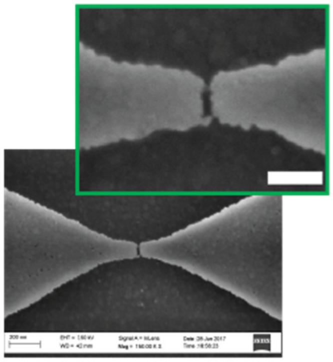

7.

<u>Results from I-V:</u> **direct and F-N tunneling**

<u>Results from SEM:</u> **1 pinched ligament**

<u>Correlation between I-V and SEM:</u> **Good**

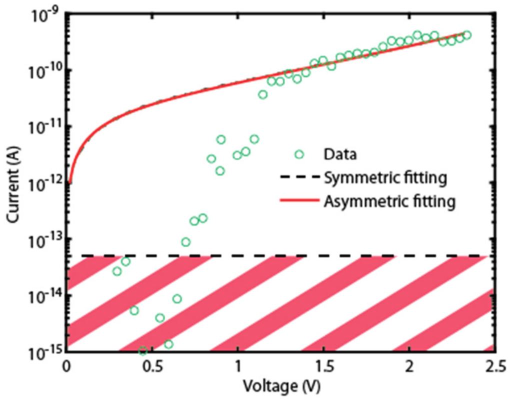

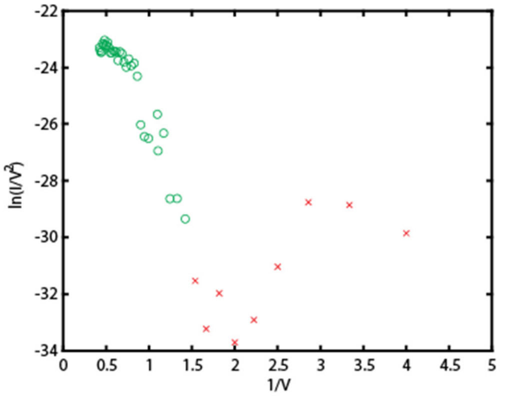

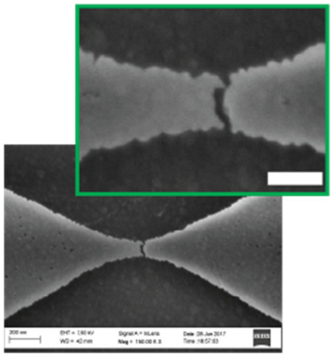

8.

<u>Results from I-V:</u> **F-N tunneling**

<u>Results from SEM:</u> **1 pinched ligament**

<u>Correlation between I-V and SEM:</u> **Good**

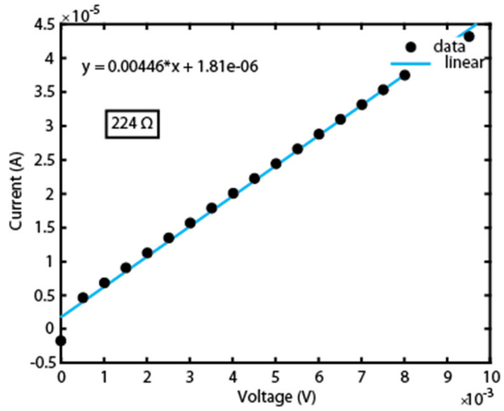

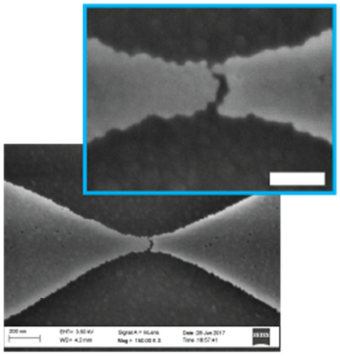

9.
<u>Results from I-V:</u> ohmic

<u>Results from SEM:</u> 1 connected ligament

<u>Correlation between I-V and SEM:</u> Good

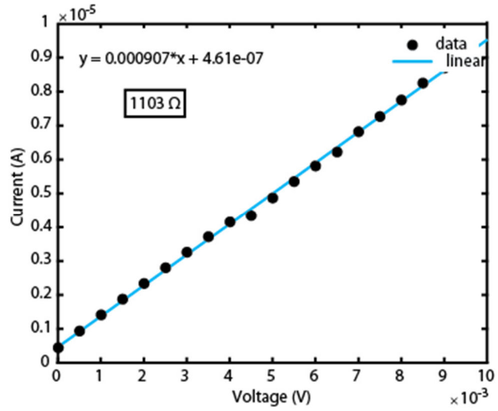

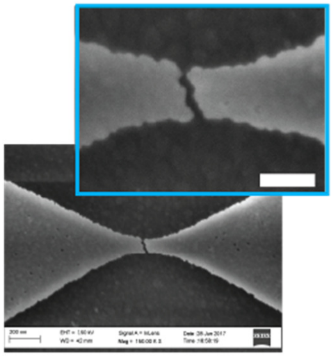

10.
<u>Results from I-V:</u> ohmic

<u>Results from SEM:</u> 1 broken ligament

<u>Correlation between I-V and SEM:</u> Poor, ligament likely melted during image acquisition

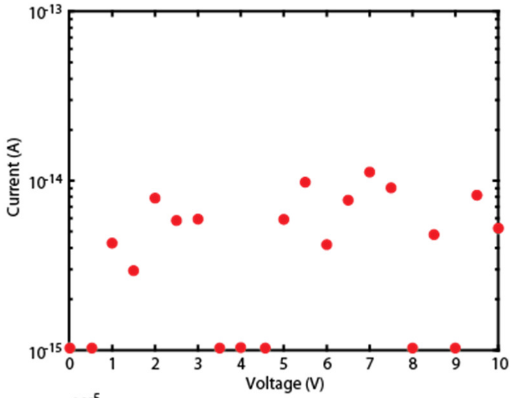

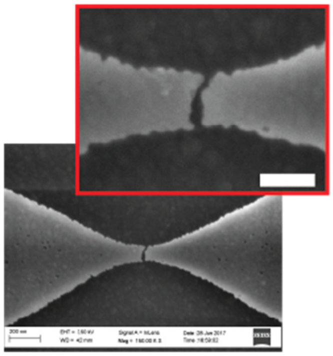

11.
<u>Results from I-V:</u> gap wider than 3 nm

<u>Results from SEM:</u> sub-10 nm gap

<u>Correlation between I-V and SEM:</u> Good

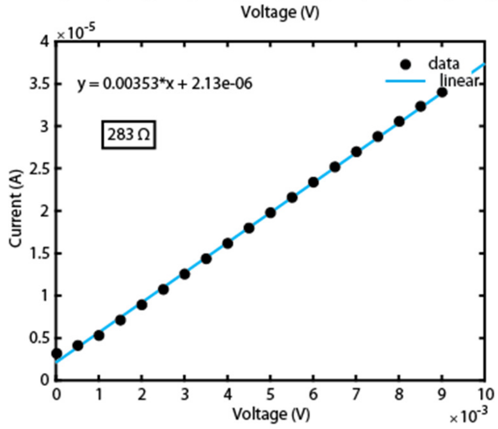

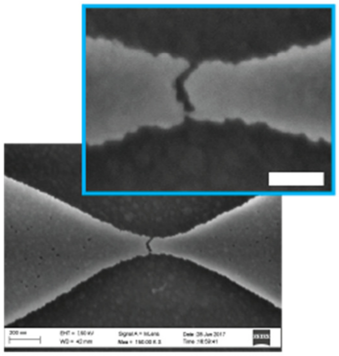

12.
<u>Results from I-V:</u> ohmic

<u>Results from SEM:</u> 1 connected ligament

<u>Correlation between I-V and SEM:</u> Good

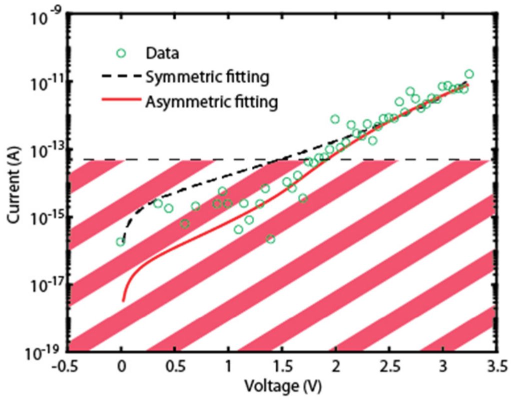

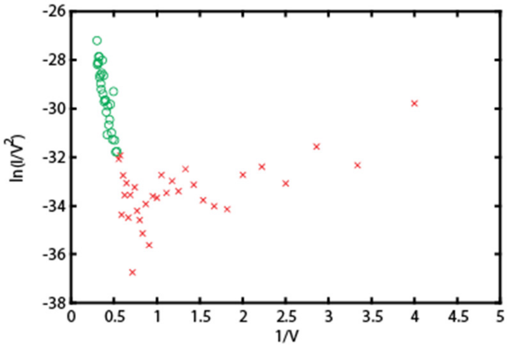

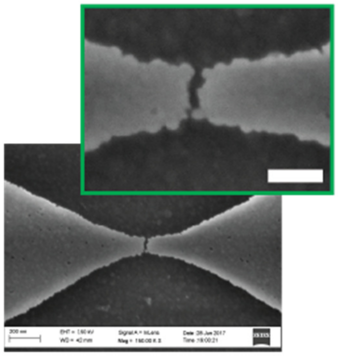

13.

<u>Results from I-V:</u> F-N tunneling

<u>Results from SEM:</u> 1 pinched ligament

<u>Correlation between I-V and SEM:</u> Good

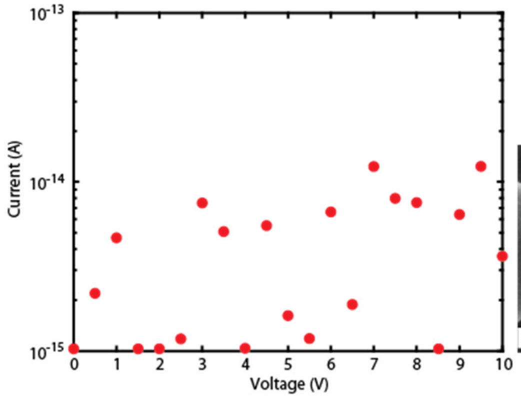

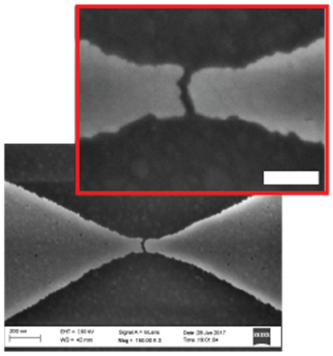

14.

<u>Results from I-V:</u> gap wider than 3 nm

<u>Results from SEM:</u> ~15 nm gap

<u>Correlation between I-V and SEM:</u> Good

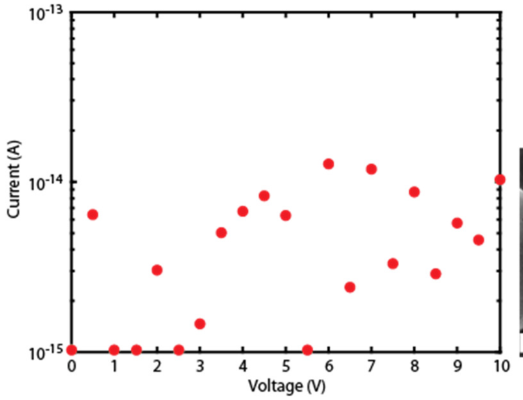

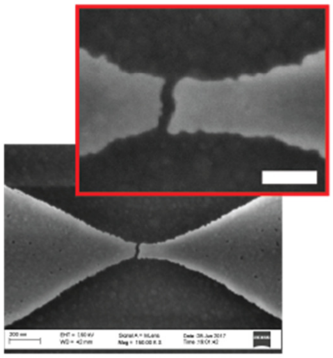

15.

<u>Results from I-V:</u> gap wider than 3 nm

<u>Results from SEM:</u> ~20 nm gap

<u>Correlation between I-V and SEM:</u> Good

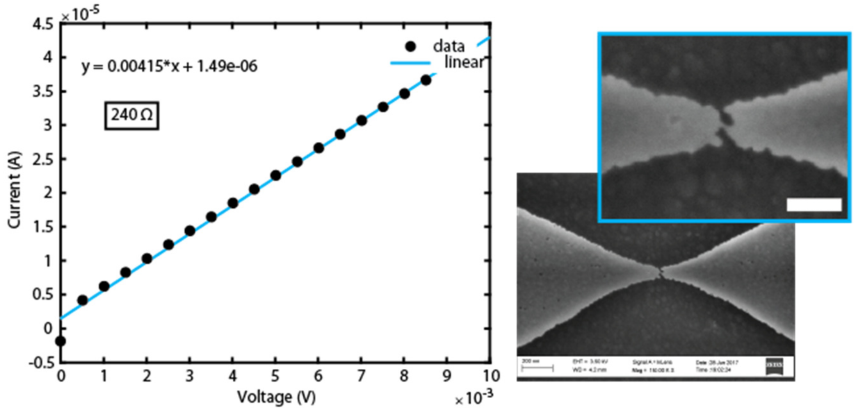

16.
<u>Results from I-V:</u> ohmic

<u>Results from SEM:</u> 1 connected ligament

<u>Correlation between I-V and SEM:</u> Good

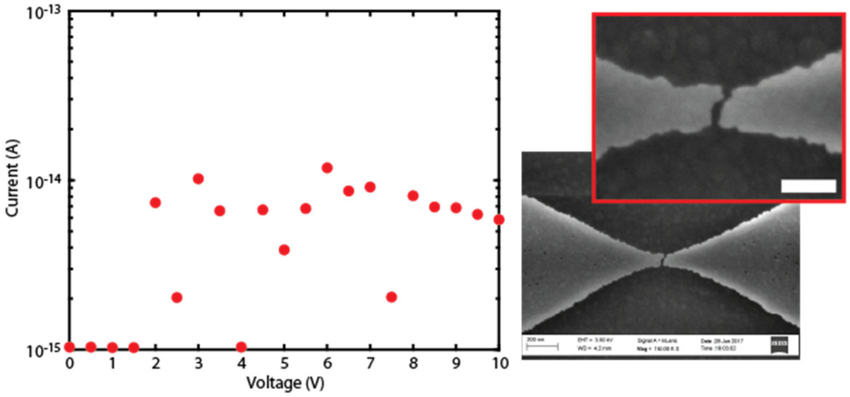

17.
<u>Results from I-V:</u> gap wider than 3 nm

<u>Results from SEM:</u> sub-10 nm gap

<u>Correlation between I-V and SEM:</u> Good

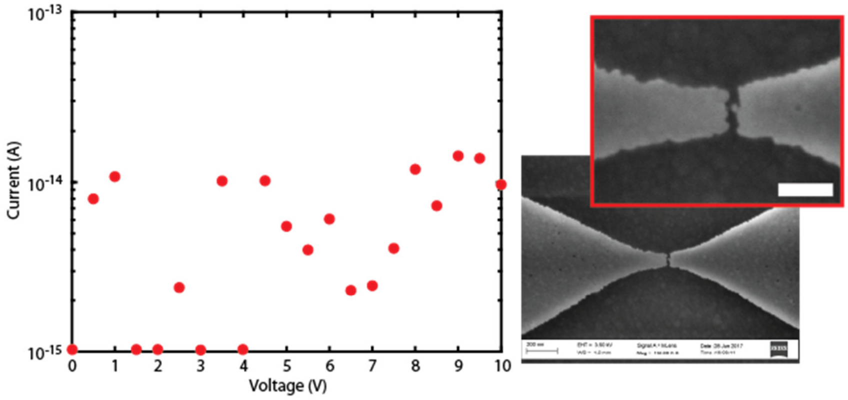

18.
<u>Results from I-V:</u> gap wider than 3 nm

<u>Results from SEM:</u> sub-10 nm gap

<u>Correlation between I-V and SEM:</u> Good

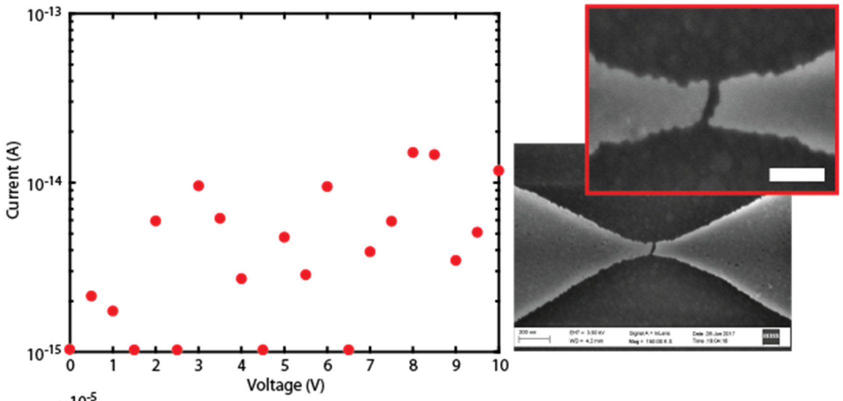

19.
<u>Results from I-V:</u> **gap wider than 3 nm**

<u>Results from SEM:</u> **~15 nm gap**

<u>Correlation between I-V and SEM:</u> **Good**

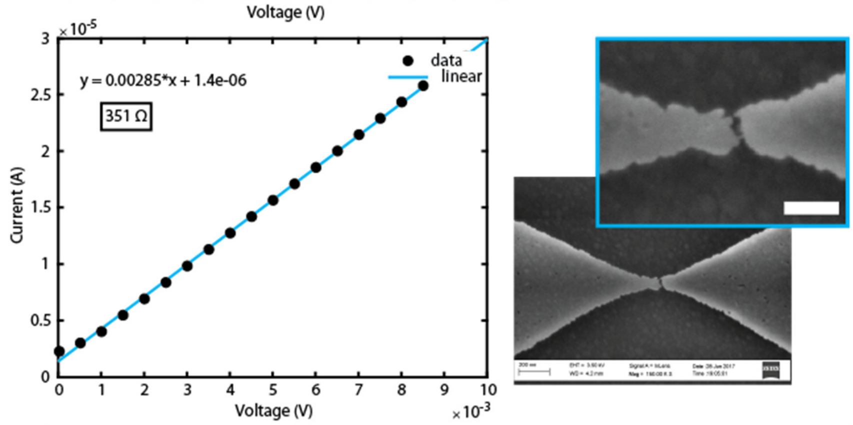

20.
<u>Results from I-V:</u> **ohmic**

<u>Results from SEM:</u> **1 connected ligament**

<u>Correlation between I-V and SEM:</u> **Good**

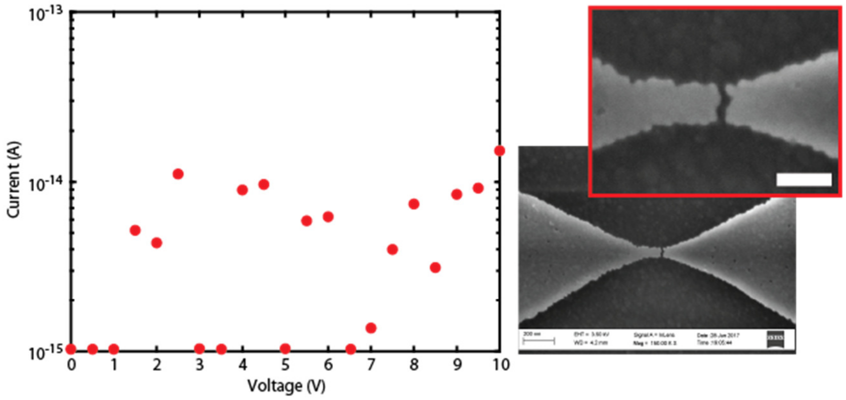

21.
<u>Results from I-V:</u> **gap wider than 3 nm**

<u>Results from SEM:</u> **~15 nm gap**

<u>Correlation between I-V and SEM:</u> **Good**

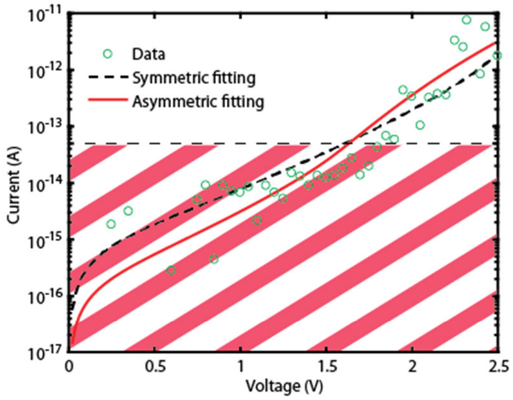

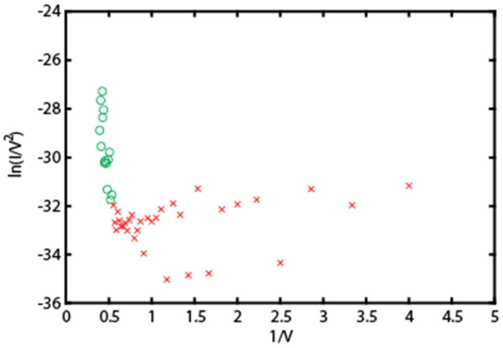

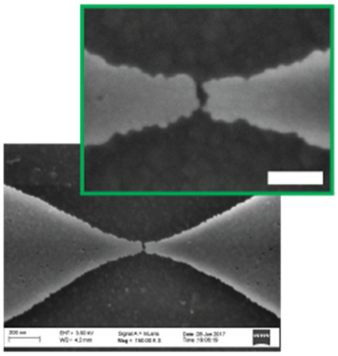

22.

__Results from I-V:__ F-N tunneling

__Results from SEM:__ 1 pinched ligament

__Correlation between I-V and SEM:__ Good

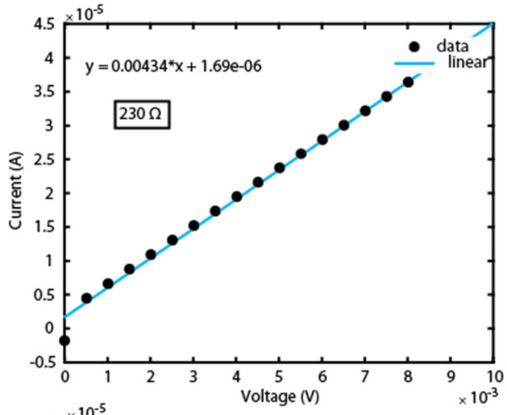

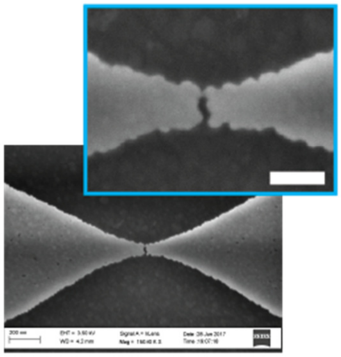

23.

__Results from I-V:__ ohmic

__Results from SEM:__ 1 connected ligament

__Correlation between I-V and SEM:__ Good

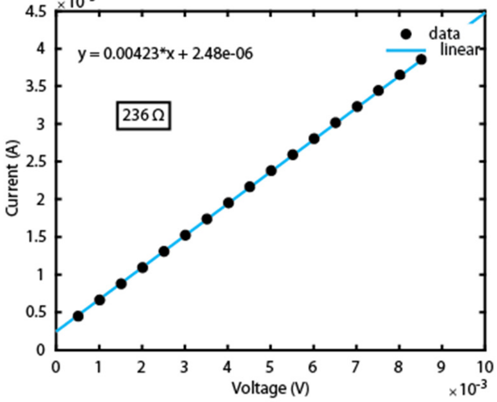

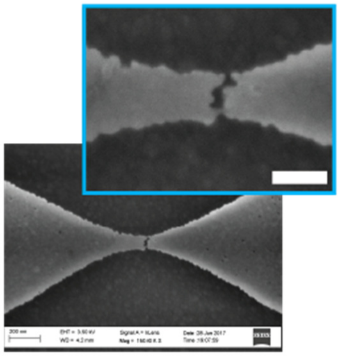

24.

__Results from I-V:__ ohmic

__Results from SEM:__ 1 connected ligament

__Correlation between I-V and SEM:__ Good

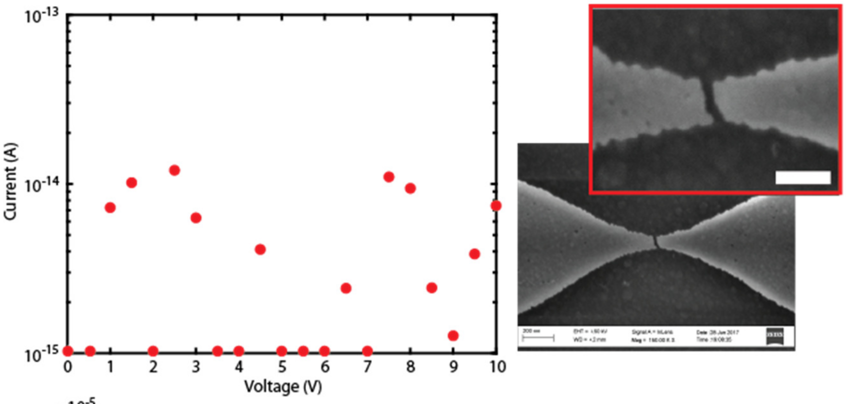

25.
<u>Results from I-V:</u> gap wider than 3 nm

<u>Results from SEM:</u> ~20 nm gap

<u>Correlation between I-V and SEM:</u> Good

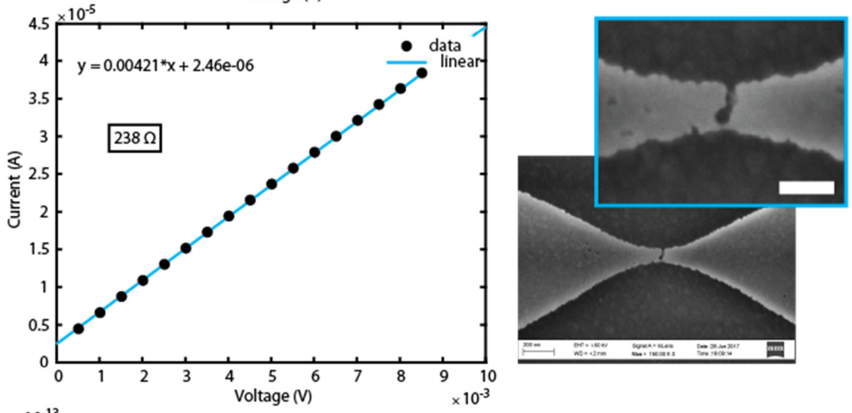

26.
<u>Results from I-V:</u> ohmic

<u>Results from SEM:</u> 1 connected ligament

<u>Correlation between I-V and SEM:</u> Good

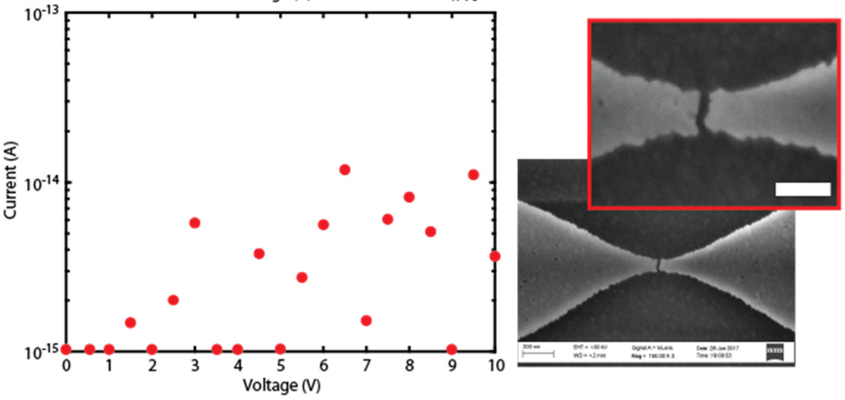

27.
<u>Results from I-V:</u> gap wider than 3 nm

<u>Results from SEM:</u> ~15 nm gap

<u>Correlation between I-V and SEM:</u> Good

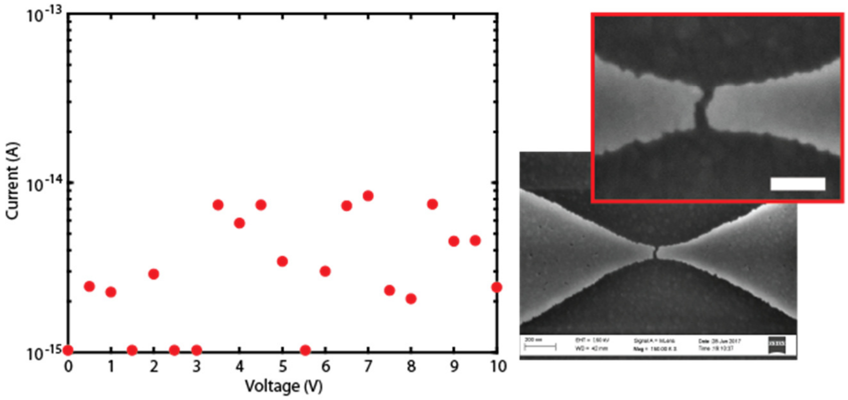

28.
<u>Results from I-V:</u> gap wider than 3 nm

<u>Results from SEM:</u> ~20 nm gap

<u>Correlation between I-V and SEM:</u> Good

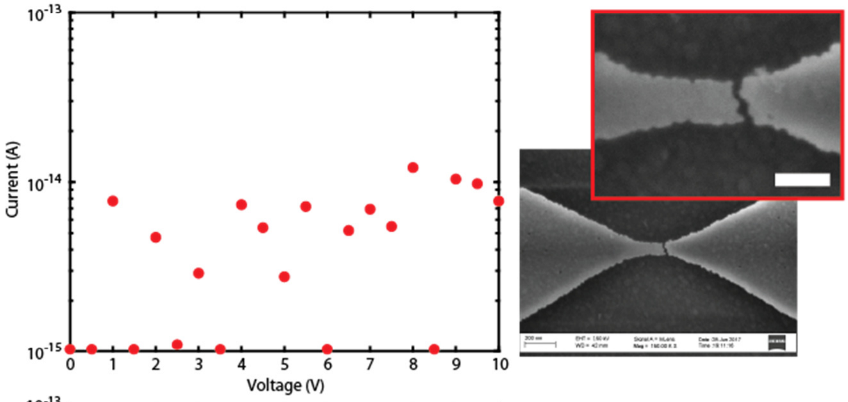

29.
<u>Results from I-V:</u> gap wider than 3 nm

<u>Results from SEM:</u> sub-10 nm gap

<u>Correlation between I-V and SEM:</u> Good

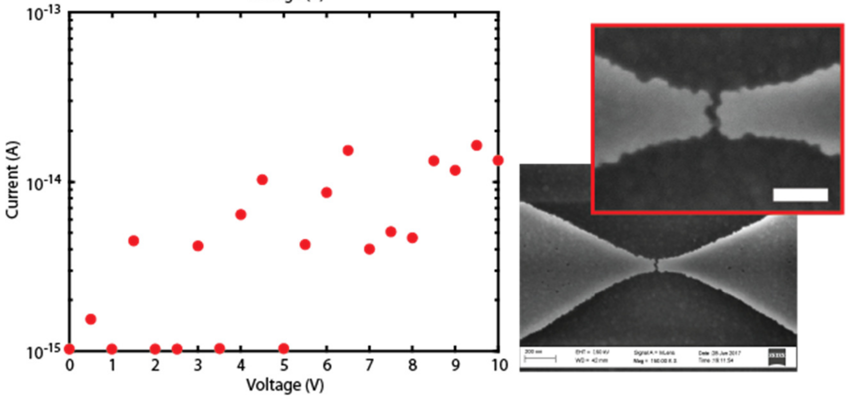

30.
<u>Results from I-V:</u> gap wider than 3 nm

<u>Results from SEM:</u> ~15 nm gap

<u>Correlation between I-V and SEM:</u> Good
